# Consumption of Alcohol Beverages and Binge Drinking Among Pregnant Women Aged 18–44 Years — United States, 2015–2017

**DOI:** 10.15585/mmwr.mm6816a1

**Published:** 2019-04-26

**Authors:** Clark H. Denny, Cristian S. Acero, Timothy S. Naimi, Shin Y. Kim

**Affiliations:** ^1^Division of Congenital and Developmental Disorders, National Center on Birth Defects and Developmental Disabilities, CDC; ^2^Maximus Federal, Atlanta, Georgia; ^3^Section of General Internal Medicine, Boston Medical Center, Boston, Massachusetts.

Drinking alcohol during pregnancy can cause fetal alcohol spectrum disorders (FASDs), including birth defects that involve central nervous system impairment, behavioral disorders, and impaired intellectual development, which can lead to difficulties with school and employment. A recent study in four U.S. communities found a 1.1%–5.0% prevalence of FASDs among first-grade students (*1*). Drinking during pregnancy might also be a risk factor for other adverse pregnancy and birth outcomes, including miscarriage and stillbirth (*2*). CDC estimated the prevalence of self-reported current drinking (at least one alcohol drink in the past 30 days) and binge drinking (consuming four or more drinks on at least one occasion in the past 30 days) among pregnant women aged 18–44 years, using 2015–2017 data from the Behavioral Risk Factor Surveillance System (BRFSS). Current drinking and binge drinking in the past 30 days were reported by 11.5% and 3.9% of pregnant women, respectively. Among pregnant women who binge drink, the average frequency of binge drinking in the past 30 days was 4.5 episodes, and the average intensity of binge drinking (the average largest number of drinks reported consumed on any occasion among binge drinkers) was 6.0 drinks. Increased implementation of evidence-based community-level and clinic-level interventions, such as universal alcohol screening and brief counseling in primary and prenatal care, could decrease the prevalence of drinking during pregnancy, which might ultimately reduce the prevalence of FASDs and other adverse pregnancy and birth outcomes.

BRFSS is a random-digit–dialed landline and cellphone telephone survey that measures behavioral risk factors from a representative sample of civilian, noninstitutionalized adults aged ≥18 years, conducted by all U.S. states and participating U.S. territories, in collaboration with CDC (https://www.cdc.gov/brfss/index.html). For this report, CDC analyzed 2015–2017 BRFSS data from 6,814 pregnant women aged 18–44 years from all 50 states and the District of Columbia. Women reported if they were currently pregnant at the time of the interview, although information about the gestational week of pregnancy was not collected. The annual median response rate* for the combined landline and cellphone sample ranged from 45.8% to 47.0%.

This report focuses on current drinking and binge drinking among pregnant women, two measures of excessive drinking^†^ in the 2015–2020 Dietary Guidelines for Americans.^§^ Respondents were asked “During the past 30 days, how many days per week or per month did you have at least one drink of any alcoholic beverage such as beer, wine, a malt beverage, or liquor?” Response choices were as follows: number of days per week, number of days in past 30 days, no drinks in past 30 days, don’t know/not sure, and refused. In addition, women respondents were asked “Considering all types of alcoholic beverages, how many times during the past 30 days did you have four or more drinks on an occasion?” Response options were as follows: number of times, none, don’t know/not sure, and refused. Finally, the intensity of binge drinking was based on the question “During the past 30 days, what is the largest number of drinks you had on any occasion?” Response choices were as follows: number of drinks, don’t know/not sure, and refused.^¶^

Prevalences and 95% confidence intervals (CIs) for current drinking and binge drinking by pregnant women were estimated overall and by sociodemographic characteristics (age group, race/ethnicity, education, employment status, and marital status). Adjusted prevalence ratios (aPRs) and CIs were calculated to examine the associations between sociodemographic characteristics and current and binge drinking, while controlling for other characteristics. Finally, frequency and intensity of binge drinking were estimated for all pregnant women who reported binge drinking. Data were weighted to represent state-level population estimates and aggregated to represent a nationwide estimate. Analyses were conducted using SAS (version 9.4; SAS Institute) with SUDAAN (version 11.0; RTI International) to account for the complex sampling method used in BRFSS.

Among pregnant women, the prevalences of reported current drinking and binge drinking in the past 30 days were 11.5% and 3.9%, respectively (Table). The prevalence of current drinking among pregnant women who were not married (15.2%) was nearly double that among those who were married (8.6%; aPR = 2.2). The prevalence of binge drinking among pregnant women who were not married (6.1%) was nearly triple the prevalence among those who were married (2.2%; aPR = 2.7). Women categorized as “other, non-Hispanic,” which included American Indian/Alaska Native, Asian/Pacific Islander, and multiracial respondents, reported a significantly higher prevalence of current drinking (18.5%) than did Hispanics, who had the lowest prevalence (8.9%; aPR = 2.0). Among pregnant women who reported binge drinking in the past 30 days, the average frequency was 4.5 (CI = 3.1–5.9) episodes, and the average largest intensity was 6.0 (CI = 5.0–7.0) drinks.

**TABLE:** Estimated prevalences* and adjusted prevalence ratios (aPRs) of current drinking^†^ and binge drinking^§^ reported by pregnant women aged 18–44 years (N = 6,814), by selected characteristics — Behavioral Risk Factor Surveillance System, United States, 2015–2017

Characteristic	Current drinking		Binge drinking	
	% (95% CI)	aPR^¶^ (95% CI)	% (95% CI)	aPR^¶^ (95% CI)
**Overall**	11.5 (10.1–13.0)	––	3.9 (3.1–4.8)	––
**Age group (yrs)**				
18–24	11.4 (9.1–14.3)	0.7 (0.5–1.0)	5.8 (4.2–7.9)	1.6 (0.8–3.1)**
25–29	9.6 (7.4–12.4)	0.7 (0.5–0.9)	3.7 (2.3–6.0)**	1.1 (0.5–2.4)**
30–34	11.6 (8.8–15.2)	0.8 (0.6–1.2)	2.6 (1.7–4.0)**	0.8 (0.4–1.6)**
35–44	14.1 (11.1–17.7)	Referent	3.1 (1.9–5.2)**	Referent
**Race/Ethnicity**				
White, non-Hispanic	10.7 (9.2–12.3)	1.3 (0.9–1.8)	3.4 (2.6–4.5)	1.1 (0.6–2.0)
Black, non-Hispanic	14.0 (10.1–19.1)	1.3 (0.8–2.1)	NA^††^	NA^††^
Hispanic	8.9 (6.3–12.3)	Referent	3.5 (2.2–5.6)**	Referent
Other, non-Hispanic	18.5 (12.6–26.3)	2.0 (1.2–3.5)	5.1 (3.1–8.6)**	1.7 (0.8–3.5)**
**Education**				
High school diploma or less	10.4 (8.0–13.2)	Referent	4.1 (2.9–5.7)	Referent
Some college	11.6 (9.2–14.6)	1.2 (0.8–1.6)	3.9 (2.5–6.1)**	1.0 (0.6–1.9)**
College degree	12.7 (10.9–14.9)	1.4 (1.0–2.0)	3.6 (2.7–4.9)	1.5 (0.8–2.8)
**Employment status**				
Employed	12.6 (10.9–14.4)	1.2 (0.9–1.5)	4.3 (3.4–5.5)	1.3 (0.8–2.3)**
Not employed	10.0 (7.8–12.7)	Referent	3.3 (2.2–4.9)**	Referent
**Marital status**				
Married	8.6 (7.1–10.3)	Referent	2.2 (1.5–3.4)**	Referent
Not married	15.2 (12.8–18.0)	2.2 (1.6–3.0)	6.1 (4.8–7.7)	2.7 (1.4–5.3)**

**Abbreviations:** CI = confidence interval; NA = not available.

* Percentages weighted to represent nationwide estimates of the U.S. population.

^†^ Defined as having consumed at least one alcohol drink in the past 30 days.

^§^ Defined as having consumed four or more alcohol drinks on one occasion at least once in the past 30 days.

^¶^ Model includes age, race/ethnicity, education, employment status, and marital status.

** Estimate might be unstable because the relative standard error is 0.2–0.3.

^††^ Estimate suppressed because the relative standard error is >0.3.

## Discussion

During 2015–2017, approximately one in nine pregnant women reported drinking alcohol in the past 30 days, and among those, about one third reported binge drinking. High blood alcohol concentrations among pregnant women might be particularly harmful to the brain of a developing fetus (*3*) and could occur even before pregnancy is recognized (*4*). A study using data from the Pregnancy Risk Assessment Monitoring System (https://www.cdc.gov/prams/index.htm) found that women who binge drink before pregnancy are more likely to drink and binge drink during pregnancy than are women who do not binge drink before pregnancy (*4*).

The overall estimates of current drinking and binge drinking among pregnant women were slightly higher during 2015–2017 (11.5% and 3.9%, respectively) than were the estimates during 2011–2013 (10.2% and 3.1%, respectively) (*5*). Although the frequency of binge drinking among pregnant women during 2015–2017 (4.5 episodes) was similar to that in the 2011–2013 BRFSS report (4.6 episodes), the intensity estimate for the 2015–2017 report (6.0 drinks) was lower than that in the earlier report (7.5 drinks) (*5*). The higher prevalences of current drinking and binge drinking among pregnant women who are not married compared with the prevalences among married women might be related to the financial stress associated with being the sole provider as well as lack of social support (*6*).

The findings in this report are subject to at least five limitations. First, data are self-reported and therefore subject to recall and social desirability biases, likely leading to underreporting of alcohol consumption during pregnancy (*7*). Second, the estimates might be affected by selection bias because the median response rates were less than 50% for all 3 years of the survey. Third, some prevalence and prevalence ratio estimates were suppressed, or flagged as possibly being unstable, because of relatively large standard errors. Fourth, pregnancy status might be inaccurate or underestimated because some pregnancies might not have been recognized at the time of interview (*8*). The percentage of currently pregnant women who reported drinking in the past 30 days and before they were pregnant likely is small because the mean gestational age of pregnancy awareness is 5.5 weeks (*8*). Finally, information on trimester of pregnancy was not available. The prevalence of drinking in pregnancy varies by trimester and is higher in the first trimester than in the second and third trimesters (*9*).

The Community Preventive Services Task Force** recommends several community-level interventions to reduce excessive drinking, such as regulating alcohol outlet density (the number of physical locations where alcohol is sold within a geographic area) through zoning and business licensing or state alcohol control agencies, implementing commercial host liability laws, and maintaining limits on hours and days of sale. The U.S. Preventive Services Task Force recommends screening and brief behavioral counseling in primary care settings for all adults aged ≥18 years, including pregnant women, to reduce unhealthy alcohol use, which includes any alcohol use by pregnant women (*10*). An American College of Obstetricians and Gynecologists Committee Opinion^††^ recommends alcohol use screening for all women seeking obstetric-gynecologic care, including counseling patients that there is no known safe level of alcohol use during pregnancy, and recommends that women who are pregnant or who might be pregnant be advised to avoid alcohol use. The combination of evidence-based community-level interventions and alcohol screening and brief counseling might decrease alcohol consumption during pregnancy, and ultimately the prevalence of FASDs, as well as other adverse pregnancy and birth outcomes.

**Summary**

**What is already known about this topic?**

Drinking alcohol while pregnant can cause miscarriage, stillbirth, and fetal alcohol spectrum disorders. There is no known safe level of alcohol use during pregnancy.

**What is added by this report?**

Analysis of 2015–2017 Behavioral Risk Factor Surveillance System data found that 11.5% of pregnant women reported current drinking, and 3.9% reported binge drinking during the past 30 days. Women who were not married were more likely to drink alcohol and binge drink during pregnancy than were married women.

**What are the implications for public health practice?**

Efforts to expand implementation of community-level interventions and universal alcohol screening and brief counseling might decrease the prevalence of drinking during pregnancy.

## Footnotes

* Calculated using the American Association for Public Opinion Research guidelines. The response rate is the number of respondents who completed the survey as a proportion of all eligible and likely eligible persons.

† Excessive drinking by women includes binge drinking (four or more drinks per occasion for women), heavy drinking (more than one drink per day on average for women), any drinking by pregnant women, and drinking by women aged <21 years. https://www.cdc.gov/alcohol/fact-sheets/prevention.htm.

§ https://health.gov/dietaryguidelines/2015/.

¶ https://www.cdc.gov/brfss/questionnaires/pdf-ques/2017_BRFSS_Pub_Ques_508_tagged.pdf.

** https://www.thecommunityguide.org/topic/excessive-alcohol-consumption.
